# Versatile lifestyles of *Edwardsiella*: Free-living, pathogen, and core bacterium of the aquatic resistome

**DOI:** 10.1080/21505594.2021.2006890

**Published:** 2021-12-30

**Authors:** Ka Yin Leung, Qiyao Wang, Xiaochang Zheng, Mei Zhuang, Zhiyun Yang, Shuai Shao, Yigal Achmon, Bupe A. Siame

**Affiliations:** a Biotechnology and Food Engineering Program, Guangdong Technion – Israel Institute of Technology, Shantou, China; b Faculty of Biotechnology and Food Engineering, Technion – Israel Institute of Technology, Haifa, Israel; c State Key Laboratory of Bioreactor Engineering, East China University of Science and Technology, Shanghai, China; d Shanghai Engineering Research Center of Marine Cultured Animal Vaccines, Shanghai, China; e Shanghai Collaborative Innovation Center for Biomanufacturing, Shanghai, China; f Department of Biology, Trinity Western University, Langley, British Columbia, Canada

**Keywords:** *Edwardsiella*, pathogen, free-living, aquatic resistome, virulence, antibiotic resistance genes, antibiotic resistant bacteria

## Abstract

*Edwardsiella* species in aquatic environments exist either as individual planktonic cells or in communal biofilms. These organisms encounter multiple stresses, include changes in salinity, pH, temperature, and nutrients. Pathogenic species such as *E. piscicida*, can multiply within the fish hosts. Additionally, *Edwardsiella* species (*E. tarda*), can carry antibiotic resistance genes (ARGs) on chromosomes and/or plasmids, that can be transmitted to the microbiome via horizontal gene transfer. *E. tarda* serves as a core in the aquatic resistome. *Edwardsiela* uses molecular switches (RpoS and EsrB) to control gene expression for survival in different environments. We speculate that free-living *Edwardsiella* can transition to host-living and vice versa, using similar molecular switches. Understanding such transitions can help us understand how other similar aquatic bacteria switch from free-living to become pathogens. This knowledge can be used to devise ways to slow down the spread of ARGs and prevent disease outbreaks in aquaculture and clinical settings.

## Introduction

The genus *Edwardsiella* is abundant in aquatic environments worldwide. The five species include three fish pathogens (*E. piscicida, E. ictulari*, and *E. anguillarum*), one environmental isolate (*E. tarda*), and one less characterized species (*E. hoshinae*) [1–4]. The three fish pathogens are also important pathogens of terrestrial animals, such as mammals, reptiles, and birds [1,4–6]. These bacteria can live freely in freshwater, estuary, and marine environments. *Edwardsiella* species carry important genes required for survival in the aquatic environments, such as genes for biofilm formation and those for adaptation to low pH, salt and oxidative stress, and low nutrients [7–10]. Additionally, the three pathogenic species of *Edwardsiella* carry important virulence genes, such as type III, type IV, and type VI secretion systems (T3SS, T4SS, and T6SS), antibiotic resistance genes (ARGs), and heavy metal resistance genes [4,11–15]. Some of these genes can be transferred to and shared with other aquatic microbiota using mobile genetic elements (MGEs) [13,14,16]. *Edwardsiella* is therefore an excellent model organism for studying horizontal gene transfer (HGT) among aquatic microbes and for host-pathogen interactions [12,13]. *E. piscicida* is an emerging pathogen and has potential to acquire extra virulent genes, such as ARG, to become highly resistant to most antibiotics (superbug). On the other hand, *E. tarda* can serve as an indicator organism for monitoring the accumulation of ARGs in the aquatic environments.

Studies on *Edwardsiella* have focused on bacterial isolates from fish during disease outbreaks on aquaculture farms. The main species isolated from diseased fish are *E. piscicida, E. ictaluri*, and *E. anguillarum* [2,12]. Many of these isolates have come from disease outbreaks in Asia, USA, and Europe [12]. Some strains have been characterized further in different fish host models to determine their virulence properties, host-pathogen interactions, and for use in potential vaccine development using molecular biology and genomics studies [6,15,17–21].

There are few reports that describe the free-living aspects of *Edwardsiella* species (i.e. *E. tarda*) in the aquatic environments. Studies are required to elucidate the water-living properties and to investigate the presence of ARGs, especially in those found on chromosomes. Many important questions remained unanswered about the organism’s adaptation to the aquatic environments as well as the major differences between free-living isolates and those isolated from diseased hosts. For example, what are the common free-living *Edwardsiella* species in the environments? Can free-living isolates in aquatic environments cause diseases, if given the opportunities? If so, what factors trigger this conversion on farms or in water? What effects do the presence or absence of hosts, sub-inhibitory or inhibitory concentrations of antibiotics and other environmental stresses, have on virulence or environmental adaptation? *Edwardsiella* is also a potential emerging pathogen of animals and humans and a potential hyper-spreader of ARGs in the aquatic resistome. It is possible that the overuse of antibiotics in hospitals and farms, the versatility of free-living *Edwardsiella* in the aquatic environment, and the multi-drug resistant phenotype, may accelerate and select this genus to become a superbug.

Many previous reviews on *Edwardsiella* have focused on its pathogenicity in fish, emphasizing its pathology and diagnosis, treatment, and prevention [1,5]. This review complements and seeks to expand previous reports to address *Edwardsiella*’s versatility to exploit different growth environments. We focused on the work done on one disease causing species, namely *E. piscicida* (other disease causing species are *E. ictaluri*, and *E. anguillarum*), and complimented this with work done on the free-living species, *E. tarda*, based on recent phylogenetic classification [2–4]. Many papers do not use the updated classification scheme to correctly type the species and to distinguish between *E. tarda* and *E. piscicida*. The majority of the *Edwardsiella* literature (~70% of the literature based on PubMed searches) are on these two species. However, this bias does not affect the conclusions in this review because our hypotheses and speculations are based on both species. We did not attempt to correct the species names in the cited papers due to insufficient information and thus gave a general genus name to represent the two combined species. However, species names were used if the cited papers correctly identified the species name. We also cited some studies from *E. ictaluri* and *E. anguillarum* to strengthen the discussion.

**Figure 1.**
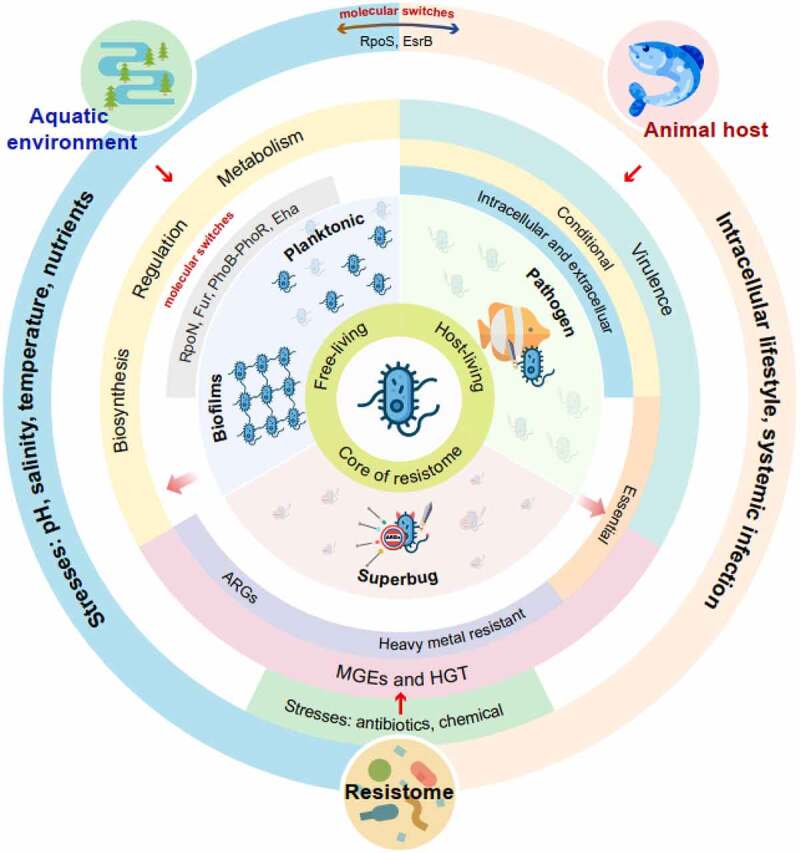
*Edwardsiella*, a water-living bacterium that adapts to free- and host-living lifestyles and is core to the aquatic resistome. (ARGs = antibiotic resistance genes; HGT = horizontal gene transfer; MGEs = mobile genetic elements).

This review focuses on *Edwardsiella* in three niche areas (Figure 1). (i) Aquatic environments: here, free-living, or communal bacteria are exposed to stresses imposed by freshwater and marine environments, such as low to mid-range temperatures changes, salinity, low nutrients, and other physical factors. (ii) Antibiotic contaminated environments: bacteria in aquaculture farms and fish hosts encounter sub-inhibitory or inhibitory antibiotic concentrations. (iii) Animal host environments: pathogenic bacteria use virulent genes to attach, invade, colonize host cells, and to spread within the host (systemic infections) [22]. In this review, we emphasize that conditional essential genes are for virulence in different tissues and hosts; some of these genes overlap with those required for free-living.

Examining the behavior of *Edwardsiella* grown in different environments may reveal differential gene expression required to overcome or exploit the changing environments. Some of these genes are interconnected and/or expressed in more than one environmental niche. We want to show how this water-living pathogen adapts its life cycle to survive and multiply in different environments. This information can be used to come up with novel ways to protect the aquatic environments, prevent the emergence of superbugs and the spread of ARGs, reduce diseases of animals and humans, and slow down the development of the aquatic resistome. We also speculate on the conversion or micro-evolution of free-living environmental isolates to disease-causing isolates and vice versa. No direct evidence can be drawn from the literature to support the above statement, but this is an important question to address in the future studies.

## *Edwardsiella* as a free-living aquatic bacterium

The genus *Edwardsiella* have developed different mechanisms to overcome rapid changes occurring naturally or anthropogenic in the aquatic environments. Naturally occurring events include changes in salinity, pH, nutrients, temperature, and other physical factors. Anthropogenic changes vary in nature and may involve direct contamination by chemical wastes (sub-lethal or lethal concentrations), such as heavy metals and antibiotics residues. These contaminants may change the pH or affect nutrient availability in water. Other anthropogenic changes may be slower and global in nature, such as temperature shift and climate change. Studies have shown that *Edwardsiella* species, such as *E. piscicida* and *E. ictaluri*, employ diverse mechanisms to overcome different stresses in the aquatic environments [9,12]. These bacteria employ internal molecular “tools” and/or adopt additional foreign genes or gene clusters from the aquatic microbiome to survive. The different genes employed by *Edwardsiella* species for water-living are described in Figure 1. These include genes involved in biosynthesis, metabolic, regulatory, virulence, and other pathways. For example, the RpoS protein of *E. piscicida* controls genes for aquatic free-living and are interconnected with virulent genes required to live inside the host [23]. Biofilm formation allows bacteria to respond and adapt to a multiple of stresses imposed on the natural aquatic environments. Biofilm formation is a useful survival phenotype and that is easily quantified in the laboratory [24]. Mechanisms to tolerate these stresses may have evolved more rapidly in *Edwardsiella* because of the need to live in the aquatic environments as a free-living microbe.

### Adaptation to salinity and pH shifts

Most bacteria either live in freshwater or in marine environments; bacteria rarely transition between the two habitats [25,26]. Switching between freshwater and marine environments is considered an evolutionary demanding process [27]. Interestingly, *Edwardsiella* species in aquatic environments have the ability to live in both freshwater and marine fish [28].

Studies have shown that some genes that promote survival of *Edwardsiella* are activated at elevated salt concentrations. For example, in *E. piscicida*, the twin-arginine translocation (Tat) system involved in the translocation of folded proteins to the periplasm, plays important roles in enabling bacteria to survive in different environments such as high temperature, high-salt conditions, and the presence of ethanol and detergents [29]. Many other genes allow aquatic bacteria to cope with multiple environmental stresses. For example, RpoN, a sigma factor, is positively regulated with oxidative stress, osmotic stress, and acid resistance [7]. RpoS is another sigma factor that binds to the RNA polymerase to directly or indirectly control expression of more than 700 genes in *E. piscicida* [23]. This common molecular switch enables bacteria to grow in different environments, including free-living (as planktonic cells or as biofilms) and host-living (extracellular and intracellular). The O-antigen ligase gene *waaL*, part of the lipopolysaccharide (LPS) biosynthesis cluster, also plays a role in *E. tarda*’s ability to tolerate high salt concentrations [30]. Additionally, Akgul *et al*. [9,31] showed that several specific universal stress proteins in *E. ictaluri* were dominant at a low pH stress and affected virulence. Specifically, the *usp*13 gene for an extra-cytoplasmic adaptor protein (CpxP)-like protein, showed a 34-fold increase under low pH stress conditions [9]. Some virulent genes in *E. piscicida*, such as *trxH* (encoding a thioredoxin) and *hutZ* (for host adaptation), were also involved in acid tolerance and biofilm formation [10,32]. Furthermore, acidic pH and low phosphate concentration signaled to *E. ictaluri* to turn on a T3SS for intracellular living [33].

### Adaptation to temperature changes

Fish disease outbreaks such as edwardsiellosis are closely associated with temperature fluctuations brought about by seasonal temperature shifts and onset of the rainy seasons in some areas. Temperature shifts in the aquatic environment can affect physical and biological factors such as dissolved oxygen content and nutrient availability. For example, incubating *Edwardsiella* in seawater at 4°C transformed the bacteria to a viable but nonculturable state [34]. This change was reversed (back to culturable) when the bacteria was transferred to a rich medium with elevated temperatures. Therefore, temperature changes can significantly impact the physiology and behavior of *Edwardsiella*. Unfortunately, no detailed studies have focused on the effect of temperature shifts on the behavior of *Edwardsiella* in an aquatic environment. Many studies have used temperature as a phenotype to characterize *Edwardsiella* mutants. For example, a *tat* deletion mutant of *E. piscicida* was more susceptible to diverse environmental stresses that included high temperature [29].

In another approach, Wei *et al*. [35] used a genome-wide transposon insertion site sequencing (TIS-Seq) analysis to examine the fitness of *E. piscicida* mutants grown in seawater at 16°C (to simulate autumn temperatures) and 28°C (to simulating summer temperatures). The authors identified genes that were up- or down-regulated at 28°C. At least 36 genes were said to be involved in *E. piscicida* adaptation to temperature changes in seawater [35]. These included 13 metabolic genes, such as those involved in oxidative phosphorylation (*nuo*) and 3 ubiquinone biosynthesis (*ubi*) genes, that were required for survival at 28°C seawater. Virulent genes, such as 10 T3SS-linked genes (including *esrA, esrB, esa*), increased the fitness of *E. piscicida* to survive in seawater at 16°C. These results suggest that adaption to water temperature fluctuations is not only related to metabolism but to virulence as well. Although electron transport and oxidative phosphorylation were essential to seawater-living at higher temperatures, virulent genes were a fitness burden to *Edwardsiella* at lower temperatures. Liu *et al*. [36] reported that EsrB controls ~990 genes, including virulence genes (T3SS and T6SS and their effectors), and genes that regulate nutrient transport (iron, amino acids, and sugars). EsrB also participates in the ROS stress response [37], metabolism, and glutamine biosynthesis [38]. Similar results were observed in free-living and pathogenic *Salmonella enterica* servovar Typhi (*S*. Typhi); genes encoding the respiratory chain such as *cyoC* and *nuoL* were required but the virulent *Salmonella* pathogenicity Island 2 (SPI-2) gene cluster (homologs of a T3SS of *Edwardsiella*) was not required and was down-regulated in water-living [39].

### Adaptation to nutrient starvation

Free-living, especially in oligotrophic water, with low levels of nutrients such as nitrate-N, phosphorus, cobalt, and iron, requires the activation of many genes required for aquatic survival [40,41]. By implication, adaptation to nutrient depletions, such as iron depletion, is not only adaptive but also an innate response for the growth of *Edwardsiella* species in water. Iron is required for many cellular processes within the bacteria and plays a key role in bacteria-host interactions. In many bacteria, the ferric uptake regulator (Fur) controls iron balance within the bacteria. Using comparative proteomics studies of a Δ*fur* mutant and its wild type in *E. piscicida*, Fur was found to up- or down-regulate at least 89 proteins and to control multiple cellular functions, including iron uptake, growth, stress resistance, metabolism, and host infection [42]. In *E. piscicida*, under iron abundant, Fur prevents transcription of iron uptake genes. In iron-depleted environments, Fur binds to and activates a Fur box in the promoters of regulated genes. Fur can also crosstalk with T3SS and T6SS genes via EsrC [43]. Deletion mutants (Δ*fur*) of *E. piscicida* showed attenuated virulence in zebrafish and turbots [8,42]. Delayed attenuation was also observed in the *fur* deletion-insertion mutant, ΔP_fur170_:TT *araC* P_araBAD,_ and the mutant was a promising live vaccine candidate [8].

### Biofilm formation

Biofilm formation is a common and natural survival mechanism in bacteria that is used to adapt to a wide range of chemical and physical stresses presented in the environments [44,45]. These stresses include salinity, pH, temperature, low nutrients, and other limitations mentioned above for water-living. Biofilm formation has been suggested to be an important growth adaptation in many natural habitats (e.g. aquatic environments) and in pathogenic niche environments (i.e. inside the hosts). Inside the biofilm, bacterial communities build an elaborate multi-layer architecture based on bacterial aggregation to protect themselves in a complex matrix of exopolysaccharides, extracellular proteins, and extracellular DNA (eDNA) [44,46]. As a result, biofilms provide a protective niche or shelter for bacteria to ensure optimal nutrients and waste products exchange, promote their survival, and increase resistance against antibiotics and other biocides. Antibiotic resistance of bacterial in biofilm can be attributed to three factors that include biochemical factors (such as antibiotic-degrading enzymes, eDNA, and quorum sensing molecules inside the matrix), molecular mechanism (such as lateral and HGT), and altered host factors (such as sub-minimal inhibitory concentration of antibiotics) [47].

Studies on *Edwardsiella* have shown that biofilm formation can be disrupted by deleting individual or multiple genes involved in different bacterial functions. Thus, biofilm-related genes have dual functions: normal cellular and biofilm formation functions. The first group of genes shown to interfere with biofilm formation involve biosynthetic genes, such as those involved in the biosynthesis of the LPS (*wibT, gne*, and *ugd*) [48] and aromatic amino acids (*aroA*) [49]. Other biosynthetic genes may directly interfere with the synthesis of extracellular components needed for biofilm formation. For example, genes such as those involved in flagella (*fliA, fliC1, fliC2* and *flhDC*) [50] and fimbria (*fimA*) [51] synthesis may affect bacteria movement and attachment to surfaces. The second group of genes that may interfere with biofilm formation are those that encode regulators (*hfq, eha*, and *rcsB*) [52–54] and sigma factors (*rpoS* and *rpoN*) [7,55]. These regulators may in turn control a subset of downstream genes. For example, Eha that regulates *fimA* and *esrB* [53] and EsrB regulates genes of the T3SS and T6SS [56]. The third group of genes that affect biofilm formation are those that regulate virulence mechanisms or virulent genes. These include genes responsible for quorum sensing (*luxS*) [57], invasion (*inv*) [58], thioredoxin H (*trxH*) [32], host adaptation (*hutZ*) [10], and serine protease autotransporter (*tsh*) [59]. Additionally, EseB, a sheath-like extracellular structure connected with the needle complex of a T3SS conferred an auto-aggregation property to the surface of *E. piscicida*, and contributed to the biofilm phenotype [60,61]. Hence, biofilm formation is complicated and is probably regulated by hundreds of genes that range from housekeeping genes to virulent genes, including genes for virulence mechanisms inside the hosts or for adaptation in the aquatic environment. Biofilm formation is also an excellent measurable phenotype to study the adaptation properties and responses of aquatic bacteria to multiple stresses present in the natural habitats [24]. Thus, biofilm can be a tool to dissect the free-living lifestyle of aquatic bacteria, such as *Edwardsiella*.

## *Edwardsiella* as core of the aquatic resistome

Antibiotics are commonly used as prophylactics and for treatment during disease outbreaks in high-density aquaculture globally [62,63]. Antibiotics and chemical residues accumulate on farms and surrounding aquatic environments. Over time, accumulated antibiotics exert selective pressures that can lead to increased incidents of antibiotic resistant bacteria (ARB) and ARGs in the environment [64]. Increased ARB can accelerate the exchange of ARGs in the aquatic microbiomes by vertical and HGT [65]. For example, a survey of medically relevant microbiota in tilapia farms in Brazil found more ARB in water samples from fish ponds than in canals that supplied water to the farms [66]. Another study found more ARGs in bullfrog farms that overused antibiotics than on polyculture farms that used less antibiotics [67]. These findings suggest that antibiotic use in aquaculture farms can select for ARB and increase the risk of spreading ARGs in the environment. It is important to note that ARGs can co-select and co-transfer with other virulent genes, change the genetic makeup of the aquatic microbiome, and increase the virulence of *Edwardsiella* and other water-borne pathogens and thus pose greater risks to animal and human health.

## *Antibiotic resistance gene profile of* Edwardsiella

Antibiotic resistance in *Edwardsiella* (such as *E. piscicida* and *E. ictaluri*) is facilitated by a wide range of ARG subtypes found in the genomes. ARGs have been found on sequenced plasmids and chromosome in *Edwardsiella* species (Table 1). We used 34 sequenced plasmids and five chromosomes from the literature and the NCBI to construct an antibiotic resistance profile of *Edwardsiella* isolates from diseased fish and free-living isolates. dos Santos et al. [68] classified the most common antibiotics used in aquaculture/agriculture into 16 families (Table 1). *Edwardsiella* is resistant to all families except phosphonic acid and others. Most of the ARGs responsible for resistance in *Edwardsiella* are reported to be located on plasmids rather than the chromosomes. However, this may be due to insufficient studies of ARGs on *Edwardsiella* chromosomes. Alternatively, the presence of ARGs on plasmids may suggest conjugation and HGT as major means of antibiotic resistance gene transfer. Plasmid-borne ARGs cover 7 of the 16 antibiotic families and include *tet, aad, sul, str, dfr*, and *flo* (Table 1). The multidrug and polymyxin (*mcr*) genes are located on the chromosome (Table 1). Additionally, phenotypic results have shown that *Edwardsiella* is resistant to an additional 5 antibiotic families (Table 1), making this organism to be resistant to 14 of the 16 antibiotic families. The presence of these ARGs makes *Edwardsiella* a core member of the aquatic resistome.

**Table 1.** Antibiotic families and antibiotic resistance genes of *Edwardsiella* from the literature and NCBI

Antibiotic families^1^	ARGs in plasmids^2^ or chromosomes^3^	Antibiotics resistance from literature^5^
1. β-lactam	*bla_CMY-2_*^2^; *ampC*^3^, *ampE^3^, mrdA^3^, mepA^3^*	Amoxicillin, ampicillin, ceftriaxone, ceftiofur, cefoxitin, cefaclor
2. Aminoglycoside	*strA*^2^, *strB*^2^, *kan*^2^, *aph(3”)-Ib*^2^, *aph(6)-Ia^2,^ aph(3ʹ)-Ia*^2^, *ant(3”)-Ia*^2^, *aadA1*^2^	Streptomycin, kanamycin, spectinomycin, tobramycin, amikacin
3. Tetracycline	*tetA*^2^, *tetR*^2^, *tetD*^2^, *tetM*^2^; *tetB^3^, tetG^3^*	Tetracycline, oxytetracycline, doxycycline
4. Macrolide	*mphA*^2^	Erythromycin, spiramycin, azitromycin, oleandomycin
5. Sulfonamide	*sul1*^2^, *sul2*^2^; *sulA^3^, sul1^3^*	sulfamethoxazole
6. Amphenicol	*catA3*^2^, *catA*^2^, *cat2*^2^, *floR*^2^	Florfenicol, chloramphenicol
7. Quinolone		Flumequine, oxolinic acid, novobiocin, ofloxacin, nalidixic acid, ciproflaxin
8. Trimethoprim	*dfrA5^4^, dfrA7^4^, dfrA12^4^, dfrA1*^2^, *dfrA12*^2^, *dhfr1*^2^; *dfrA12^3^*	Trimethoprim
9. Glycopeptide		Vancomycin
10. Polypeptide		Bacitracin
11. Multidrug	*emrA^3^, emrB^3^, emrD^3^, mdtC^3^, mdtI^3^, mdtJ^3^, mdtH^3^, acrD^3^, acrR^3^, mdtD^3^, tolR^3^*	
12. Polymyxin	*mcr^3^*	Colistin
13. Lincosamide		Linocomycin, clindamycin
14. Rifamycin		Rifampin

^1^16 families of antibiotics as described by Dos Santos *et al*. [68], and the last two groups not in table are: phosphonic acid and others.

^2^References for the ARG subtypes on plasmids: 13, 16, 19, 79, 100–104. Some can be only found in NCBI (https://www.ncbi.nlm.nih.gov): pEI3, NC_020280.1; pEI-MS-17-156-1, NZ_CP028814.1; pETW41, NZ_CP019441.1; pEP-S07-348-1, NZ_CM009826.1; pET-LADL88-209-2, NZ_CM009829.1. Most of the ARG subtypes are plasmid-encoded.

^3^Denotes the ARG subtypes encoded on chromosomes. The five chromosomes used are from *E. anguillarum* ET080813 (NZ_CP006664.1); *E. ictaluri* 93–146 (NC_012779.2); *E. piscicida* EIB202 chromosome (NC_013508.1); and two from *E. tarda* FL95-01 (NZ_CP011359.1) and KC-PC-HB1 (NZ_CP023706.1).

^4^Some ARG subtypes are not clearly delineated as chromosome- or plasmid-encoded.

^5^References for the ARG phenotypes 11,13,16,19,79,80,100–106.

Most ARGs studies are based on a few novel plasmids isolated from *E. ictaluri* and *E. piscicida* infected fish (Table 1). Future work should focus on characterizing other ARGs found on the plasmids of free-living *E. tarda* isolates found in the aquatic environment. Additional work to characterize the many multi-drug resistant genes, some with unknown functions and found on the chromosomes, is required to build a complete ARGs profile of *Edwardsiella*. For example, colistin resistance genes (*mcr*) are mostly located on the *Edwardsiella* chromosomes but it is not clear if they have other functions (Table 1). We speculate that the totality of ARGs from the plasmids and chromosomes may benefit *Edwardsiella* in the aquatic environments in addition to enhancing the virulence of the pathogenic types. Since plasmids can easily be lost due to fitness costs, integration of ARGs into the bacterial chromosome may offer the bacterium a more permanent solution. Thus, research is required to detect and elucidate the source of ARGs in the *Edwardsiella* chromosomes, and to explain possible mechanisms for chromosomal integration. Plasmids in *Edwardsiella* should be compared with other enterics (such as *E. coli* and *Salmonella*) and other water-borne bacteria (such as *Aeromonas* and *Vibrio*) found in similar aquatic environments in order to build a complete picture of the aquatic resistome.

## *Mechanisms of antibiotic resistance in* Edwardsiella

Antibiotic resistance mechanisms can be grouped into four major categories: enzymatic inactivation/modification of antibiotics, decrease penetration and/or extruding of antibiotics through efflux pumps, changes to and/or bypassing target metabolic step, and global cell adaptive mechanisms [69]. For example, there are more than 30 different *tet* genes that confer resistance to tetracycline that use different resistance mechanisms, including efflux pumps, ribosomal protection, and enzymatic modifications [70,71].

Supply of exogenous metabolites can reverse or change antibiotic resistance in *Edwardsiella* and other Gram-negative bacteria by affecting bacterial metabolic processes such as increasing citric acid cycle flux, NADH production, proton motive force, and the expression of outer membrane proteins. For example, multidrug-resistant *E. tarda* and *E. piscicida* were shown to be more sensitive to kanamycin when supplemented with exogenous alanine and/or glucose [72]. Other studied showed that aminoglycosides given in combination with specific metabolites, such as glucose and pyruvate, increased the antibiotic sensitivities of *Escherichia coli* and *Staphylococcus aureus* [73]. A plausible explanation to the use of metabolomics (such as glucose and alanine) to aid the killing of ARB is that exogenous metabolites increase metabolic flux through the TCA cycle and thus accelerate the uptake of antibiotics [72,73]. However, Su et al. [74] showed that depressing the pyruvate cycle contributed to the acquisition of ampicillin resistance in *E. piscicida*. Other studies have also shown that *E. piscicida* can adapt to different antibiotic stresses by regulating the expression of different outer membrane proteins under different antibiotic stresses [75].

Heavy metals can also affect antibiotic resistant bacteria’s response to antibiotics. For example, a combination of antibiotics and heavy metals (i.e. cationic metals) are widely used in aquaculture as therapeutics to promote growth and control diseases [76]. However, presence of heavy metals can also assist and accelerate the evolution of antibiotic resistance on farms. Resende *et al*. [66] reported a positive correlation between ARB frequency and tolerance to cationic metals, such as nickel, zinc, and copper in tilapia farms in Brazil. In another study, Lee and Wendy [11] isolated three hundred strains of *Aeromonas hydrophila* and *E. tarda* that were resistant to several antibiotics and heavy metals on tilapia farms. It is important to note that ARGs and heavy metal resistance genes can be located on the same plasmids [76]. Therefore, cross-resistance mechanisms to antibiotics and other chemicals are possible in bacteria [76]. Thus, biofilm, exogenous metabolites, heavy metal resistant genes, and possibly other factors, can act as additional variables to influence the development of bacterial antibiotic resistance in bacteria, including *Edwardsiella*.

### HGT mechanisms of ARGs

ARB in aquatic environments or resistomes can serve as sources and reservoirs of ARGs. Different resistomes in habitats may interconnect to allow the spread and transfer of ARGs from nonpathogenic isolates to animal and human pathogens when subjected to selection pressure [64,77]. The spread of ARGs in the microbiome or resistome can be attributed to clonal spread of dominant strains (vertical gene transfer) and transfer to other species by HGT. However, it is widely accepted that HGT is the key driver in the spread of ARGs and the main driver in the evolution of resistomes [65,78]. The major HGT processes utilize conjugation involving plasmids, transductions by bacteriophages, and natural transformation by extracellular DNA (eDNA). Other mechanisms involving mobile genetic elements (MGEs) that include integrons, transposons, insertion sequences, and other mobile elements, can work hand in hand with HGT to spread ARGs and other accessory genes from one bacteria to another, or to stably anchor ARGs into the bacterial genomes [65]. In general, plasmids with ARGs have high fitness costs to the bacteria and are easily lost when the selection pressure is removed [77]. Moving ARGs and other virulent genes from the plasmid and inserting them into the bacterial chromosomes can stably maintain these genes in the resistome. Quantifying the rate and direction of gene flow of ARGs among clinical, farm, and environmental microbiomes and documenting the spread of ARGs by HGT [65] is the next frontier in the study of bacterial gene transfer.

Conjugation requires physical contact between two bacterial cells, to allow transfer of plasmids from the donor to the recipient cell. *Edwardsiella* species carries a wide range of plasmids with sizes ranging from 2 Kb to 127 Kb (e.g. p080813-2 and p080813-1 in *E. anguillarum* ET080813) [4] (Table 1). Some free-living isolates of *Edwardsiella* in the environment can house multiple plasmids, such as the five found in *E. tarda* LADL88-209 (https://www.ncbi.nlm.nih.gov). Additionally, a single *Edwardsiella* plasmid can carry multiple ARGs, such as the *tet* and *cat* operons found on the pETX plasmid of *E. piscicida* [19] and the *tetR, tetR, aph* [6]Id, *aph*(3”)-Ib, *sul2*, and *catIII* on the pEIB202 plasmid of *E. piscicida* [79]. The pETX and pEIB202 plasmids can also transfer ARGs to different bacteria genera [13,19]. Other plasmids such as the pCK41 of *E. piscicida* can carry ARGs and other virulence-associated genes [16].

Since *Edwardsiella* species that harbor plasmids with ARGs are abundant in the aquatic environment, these bacteria are ideal indicator candidates for antibiotic resistance surveillance to monitor antibiotic usage in specific habitats. Lo *et al*. [80] sampled *Edwardsiella* isolates from eel farms with diseased animals and compared them with isolates from surrounding farms in the same region. The authors found similar plasmid types in different habitats and linked conjugation to the spread of ARGs. Bacteria conjugation is considered a subdivision of T4SS [81]. Indeed, T4SS genes have been found in the *E. piscicida* pEIB202 plasmid [79] and in four *E. ictaluri* strains [14]. Liu *et al* [13] further reported that the ten T4SS genes found in pEIB202 were functional and responsible for conjugation. Furthermore, the same authors demonstrated that the plasmid pEIB202 could be transferred from *E. piscicida* to *E. coli, Vibrio alginolyticus*, and *V. harveyi* by conjugation. The plasmid could also be transferred back to *E. piscicida* from *E. coli* or *Vibrio*. More detailed studies are required to examine the mechanisms of conjugation and to test whether inhibitors of HGT can be used as novel treatments to reduce or prevent the spread of antibiotic resistance [82].

The role of transduction and transformation in HGT of ARGs and other bacterial accessory genes in the natural environments is poorly understood and their contributions to ARGs transfer are possibly underestimated [65,83]. Transduction and transformation do not require direct cell-to-cell contact between the donor and recipient. The mechanisms or processes involved are also considered to be very efficient. Bacteriophages survive in the environment for much longer than bacteria and they greatly outnumber bacteria in the aquatic environment to ensure high rates of transduction frequencies [83]. Generalized transduction is believed to play a more dominant role than specialized transduction in ARGs transfer [84]. Transduction also plays important roles in the spread of ARGs from bacterial communities in one habitat to another since the donor and recipient bacteria are not required to be present at the same time or in the same space [83].

Like bacteriophage, extracellular ARGs (eARGs) are also abundant in the aquatic environments and do not require cell-cell physical contact to bring about transformation. These eDNAs can arise by secretion from live bacterial cells or by lysis of dead cell. Transformation is believed to require a shorter time, compared to transduction, since eARGs are unprotected in the environments [65] and are abundant in many habitats, such as activated sludge from wastewater treatment plants (WWTPs) [85], aquatic sediment [86], and swine manure [87]. The concentration of functional eARGs increases in environments rich in organic or inorganic particles that protect and prevent degradation by nucleases [88]. Liu *et al*. [89] also showed that chlorination in WWTPs increased the abundance of eARGs, such as *tet, sul*, and *ampC* in wastewater effluents. These eARGs can be integrated into naturally competent, nonresistant bacteria, resulting in the proliferation of ARB [89].

## *Edwardsiella* as a pathogen of fish and other animals

In addition to free-living *Edwardsiella* in the aquatic environment, the organism can also exhibit extracellular and intracellular lifestyles within animal hosts. The bacterium can sense specific signals in its environments to activate appropriate genes required for fitness, whether in an aquatic environment, or for infection and survival in the host animal or in the host cell [90]. Here we consider the requirements for extracellular and intracellular living within the host, specifically, the conditional essential genes for host living. *E. piscicida* initiates entry into the host by first attaching to the epithelia of the gastrointestinal tract of animals or gills of fish [22]. Upon entry into the host, the bacterium evades the host innate immune system, enters, survives, and multiplies within host cells in *Edwardsiella*-containing vacuoles (ECVs), and eventually breaks out of ECVs to cause systemic infection [12,22].

## Edwardsiella *genes required for host extracellular and intracellular survival*

Pathogens infecting animal hosts encounter diverse and changing conditions that are more sophisticated than *in vitro* environments. Past studies showed that the pathogenicity of *E. piscicida* depended on both T3SS and T6SS [18,36,60]. However, there is limited knowledge about the involvement of secretion systems in the early stages of infection and whether other genes contribute to host colonization by *E. piscicida*. Most *in vivo* experiments have used the J774A.1 murine macrophage-like cells to study intracellular growth and turbot fish to study host infection [21,36,79].

Transposon insertion sequencing (TIS) has been used to explore the nature of essential genes needed to support the *in vitro* and *in vivo* growth of *E. piscicida* [36,90,91]. Yang et al. [91] used TIS technology to show that *E. piscicida* required 364, 257 and 198 genes for optimal growth in the liver, spleen, and kidney of turbot, respectively. Sixty-eight genes that play important roles in secretion systems and biosynthetic pathways of *E. piscicida* were required for growth in all three organs. The rest were organ specific and may mediate bacterial processes necessary for fitness in specific organs [91].

Wei *et al*. [90] also used TIS to compare the fitness of insertion mutations in *E. piscicida* grown in Dulbecco’s Modified Eagle’s medium (DMEM), J774A.1 cells, and turbots. The initial library was grown in LB medium before transfer to the different experimental conditions. Growth in LB medium required many genes involved in energy metabolism (e.g. *acnB* and *ace*FE), amino acid metabolism (e.g. *dspADEF*) and information processing (e.g. *dnaABEGJNX, polA, gyrAB*) [90]. However, only 52 genes, categorized as conditionally essential, were found to be important for growth in DMEM. Additionally, no genes encoding the T3SS and T6SS were required for growth in DMEM. Growth of *E. piscicida* in J774A cells on the other hand required 67 genes that included 8 T3SS genes (*esaH, esaG, esaS, esaT, esaU, esrA, esrB*, and *esrC*) and 6 oxidative phosphorylation genes (*nuoA, nuoD, nuoF, nuoI, nuoJ*, and *nuoM*) [90]. There were no gene overlaps between bacteria grown in DMEM and J774A.1 cells. These results indicated that virulence gene expression was essential for bacterial infection of the host but not for *in vitro* growth. Interestingly, the majority of T3SS (29 of 34) and all T6SS [16] genes were found to be conditionally essential in turbot [90], confirming the importance of these genes for the pathogenesis of *E. piscicida* [18,60,91]. Similar to the J774A.1 cellular environment, *E. piscicida* growth in turbot was also dependent on oxidative phosphorylation and NADH metabolism related genes [90]. Additionally, genes encoding LPS (e.g. *waaG, waaQ, waaL, waaF, waaC, walW, walR, wabH*, and *wabK*) were also required for *E. piscicida* growth in turbot [90].

Studies on conditional essential genes for *E. piscicida* fitness and virulence in hosts may lead to the discovery of new targets to generate live attenuated vaccines. Known live attenuated vaccine candidates, such as the WED strain that lacks *aroC* and T3SS components (*eseB, eseC, eseD* and *escA*), have attenuated growth in turbot [20,92,93]. Other target genes for live attenuated vaccine development include *sip2* (essential for survival in serum) [94], *ETAE_0023* (important for invasion and colonization) [95], and *evrA* (essential for activating virulence gene expression) [90]. However, live attenuated vaccine studies of *Edwardsiella* are still in the early stage of development, and conditional essential genes may provide good candidates for further exploration.

## Conclusion

In this review, we discussed *Edwardsiella*’s lifestyle from three perspectives: free-living in an aquatic environment, intracellular and extracellular host living, and as a core bacterium in the aquatic resistome. Pathogenic (disease causing) isolates of *Edwardsiella* (i.e. *E. piscicida*) behave differently in different habitats; as single planktonic cells or as communal biofilms (in aquatic environment) and extracellular or intracellular in infected hosts (such as fish). *Edwardsiella*’s behavior is like *Salmonella* in that, inside the host, it can reside in a vacuole (ECV) and eventually cause systemic infection [12]. Important questions remain on how these pathogens, including *Edwardsiella* and other water-borne pathogens such as *Vibrio* and *Aeromonas* species, regulate gene expression to survive and thrive in constantly changing habitats.

At least two molecular switches, RpoS and EsrB, have been identified in pathogenic isolates of *E. piscicida*. RpoS aids *E. piscicida* to survive and adapt to environmental stresses such as starvation, high salinity, H_2_O_2_ (reactive oxygen species, ROS) exposure, and biofilm formation [55]. RpoS allows *E. piscicida* to sense and adapt to changes in the environment. RpoS also inhibits *esrB* expression to down-regulate T3SS and T6SS [23]. In the host, this may require the organism to turn on virulent genes such as T3SS and T6SS. Similar observations have been made in *Legionella pneumophila* in which, RpoS is not only used as a global molecular switch to shut down metabolic process for survival in aquatic environments, but it can also be used to turn on virulence genes for intracellular living in protozoan and human hosts [96]. EsrB is also a global regulator and an essential response regulator in the EsrA-EsrB two-component system of *E. piscicida* [36]. Therefore, RpoS and EsrB control the balance between physiological adaptation to environmental changes and virulence for host living in pathogenic isolates of *Edwardsiella*. Similar observations have been made in *Salmonella enterica* servovar Typhimurium (*S*. Typhimurium) where SsrB (homolog of EsrB) encoded on the *Salmonella* SPI-2 pathogenicity Island, converts *S*. Typhimurium from intracellular living to biofilm formation inside the host [97]. This conversion involves phosphorylated/unphosphorylated forms that activate or silence a sub-population of genes [97].

An important question remains unanswered: how do free-living isolates of *Edwardsiella*, which do not have many virulence genes such as EsrB and T3SS/T6SS gene clusters, convert to pathogenic types and a host-living lifestyle? In other words, what other molecular switches are used in planktonic and communal biofilm forms for self-preservation in a free-living lifestyle and pathogenesis in a host-living lifestyle? For examples, can *E. tarda* use RpoN, Fur, Eha, and PhoB-PhoR to regulate growth under iron or phosphate limitation, oxidative or osmotic stress, and for acid tolerance [7,42,43].

Since nonpathogenic *Edwardsiella* (i.e. *E. tarda*) do not have many virulent genes and are normally unable to infect and grow inside fish hosts, these environmental isolates are well adapted to various stresses associated with the aquatic environments. Ferenci [98] proposed trade-offs in free-living bacteria between the need for self-preservation and nutritional competence, called SPANC balance. The SPANC balance explains how bacteria try to balance growth-related selective pressures to opposing stress-related selective pressures, to give rise to polymorphisms in porin and RpoS levels that are found in distinct bacterial strains.

Similar groups of *Edwardsiella* genes involved in biosynthesis, regulation, metabolism, and virulence are required for biofilm formation and planktonic adaptation to changes in salinity, pH, temperature, nutrient starvation. Importantly, some genes, such as those for sigma factors (*rpoN* and *rpoS*) [7,55], regulators (*fur, phoB-phoR*) [43], and virulence (*hutZ, trxH, tsh*, and genes related toT3SS and T6SS) [10,32,59], are required for aquatic free-living. Hence, some genes or gene clusters handle multiple stresses necessary for aquatic free-living. Furthermore, some genes are required for host-living (turbot) by pathogenic *Edwardsiella* and include genes for energy metabolism (*nuo*) T3SS and T6SS (*esrA, esrB, esrC*, esa, ese), and LPS biosynthesis (*waaL*) [90]. Therefore, multiple genes or gene clusters are required for host-living. Some of these genes overlap with those required for survival during temperature fluctuations, high salinity, and iron limitation [30,35,99]. It is important to note that some overlaps exist in the pathways or genes required for free-living and host living. Thus, communication via environmental cues allows *Edwardsiella* to regulate gene expression or trade-off expression profiles to support different lifestyles.

Genes required for free-living *Edwardsiella* (i.e. *E. tarda*) may constitute the essential or core genes for survival. These genes are enriched, selected for and abundant due to the fitness cost. However, when selection is applied, by antibiotics or heavy metals, the free-living isolates can pick up plasmids or ARGs from the aquatic resistome via eDNA, plasmids, bacteriophages, or MGEs for survival. Occasionally, these free-living isolates will pick up virulent and other accessory genes such as T3SS and T6SS gene clusters (pathogenicity Islands) via MGEs. These genes can be picked up from water sediments or inside the fish guts. We postulate that the number of disease-causing isolates of *Edwardsiella* (i.e. *E. piscicida*) in aquatic environments is small and cannot successfully compete with free-living isolates due to the high fitness cost associated with maintaining virulent genes. However, if the fish hosts’ immunity is weakened, due to temperature fluctuations and over-crowding, free-living isolates with newly acquired virulent genes can multiply and cause disease outbreaks. Later, when the outbreak subsides, these disease-causing isolates of *Edwardsiella* may be released into the aquatic environment. Eventually, due to fitness cost associated with maintaining virulent genes, these genes will be diluted out or lost as the isolates revert to a free-living lifestyle with very few virulent genes. This raises the question: can *E. tarda* (environmental isolates) be transformed into *E. piscicida* (diseased isolates) and vice versa? Comparative genomics and targeted transformation experiments may provide some answers to the above question.

Lastly, there are few reports in the literature on conjugation, transduction, and transformation involving ARGs in the aquatic microbiome. We propose that *E. tarda* is core to the aquatic resistome because it is abundant and carries multiple ARGs. This organism is also an ideal model organism for studying ARGs transfer in the aquatic microbiome. For example, free-living isolates carry several plasmid types with diverse origins and modes of transfer. Thus, genetic exchange studies involving *E. tarda* is the next frontier to elucidate the exchange of virulent genes between pathogenic and nonpathogenic strains in the aquatic environments. The knowledge generated from such studies will not only increase our understanding of the evolution and development of the aquatic resistome but will also help us understand the origins of pathogenic strains that cause disease outbreaks in aquaculture farms. This perhaps, will also elucidate the mechanisms involved in the development and emergence of water-borne pathogens of humans and animals.

## Abbreviations

Antibiotic resistance genes: (ARGs);
antibiotic resistant bacteria: (ARB);
extracellular DNA: (eDNA);
ferric uptake regulator: (Fur);
horizontal gene transfer: (HGT);
lipopolysaccharide: (LPS);
mobile genetic elements: (MGEs);
insertion site sequencing: (TIS);
type III secretion system: (T3SS);
type IV secretion system: (T4SS);
type VI secretion system: (T6SS)
